# The Urethral Rhabdosphincter, Levator Ani Muscle, and Perineal Membrane: A Review

**DOI:** 10.1155/2014/906921

**Published:** 2014-04-27

**Authors:** Nobuyuki Hinata, Gen Murakami

**Affiliations:** ^1^Department of Urology, Kobe University Graduate School of Medicine, 7-5-1 Kusunoki-cho, Chuo-ku, Kobe 650-0017, Japan; ^2^Department of Anatomy, Tokyo Dental College, 1-2-2 Masago, Mihama-ku, Chiba 261-8502, Japan; ^3^Division of Internal Medicine, Iwamizawa Kojin-kai Hospital, 297-13 Shibun-cho, Iwamizawa 068-0833, Japan

## Abstract

Detailed knowledge of the anatomy of the rhabdosphincter and adjacent tissues is mandatory during urologic surgery to ensure reliable oncologic and functional outcomes. To characterize the levator ani (LA) function for the urethral sphincter, we described connective tissue morphology between the LA and urethral rhabdosphincter. The interface tissue between the LA and rhabdosphincter area in males contained abundant irregularly arrayed elastic fibers and smooth muscles. The male rhabdosphincter was positioned alongside the LA to divide the elevation force and not in-series along the axis of LA contraction. The male perineal membrane was thin but solid and extends along the inferior margin or bottom of the rhabdosphincter area. In contrast, the female rhabdosphincter, including the compressor urethrae and urethrovaginal sphincter muscles, was embedded in the elastic fiber mesh that is continuous with the thick, multilaminar perineal membrane. The inferomedial edge of the female LA was attached to the upper surface of the perineal membrane and not directly attached to the rhabdosphincter. We presented new diagrams showing the gender differences in topographical anatomy of the LA and rhabdosphincter.

## 1. Introduction

The levator ani muscle (LA) and the urethral rhabdosphincter are both striated muscles. Striated muscle cells or fibers are surrounded by the endomysium comprising type IV and other collagens, whereas intramuscular and extramuscular tendons are made of type I collagen [1–4]. Intermediate filaments, such as desmin and vimentin, connect muscle myosin to collagen fibrils [5, 6]. Typical striated muscle fibers carry a series of specific collagenous structures (a form of enthesis [7]) to conduct the force of contraction to a bone or ligament via a tendon. In short, contraction force is conducted from endomyseal type IV collagen to type I collagen of the intramuscular tendon [1, 2], and the extramuscular tendon is connected to bone via the type I collagen-rich fibrocartilage [7].

Nevertheless, the LA and rhabdosphincter do not carry such a series of collagenous structures but are accompanied by abundant elastic fibers and/or smooth muscles [8–10]. Unlike the usual interface or enthesis between locomotive structures, mechanical roles of elastic fibers have been described in the vocal cord as well as the joints between ear ossicle [11–13]. In these tissues, hyaluronan seems to play a critical role in lubrication between elastic fibers. Hinata et al. [14] have also reported the distribution of hyaluronan in the urethral wall, but previous discussions of uroanatomy have dealt little with mechanical aspects. The present review reconsiders the topohistology of the pelvic floor, focusing on the roles of fibrous structures and force transduction, in addition to the topographical relationship between fibrous structures and nerves.

## 2. Materials and Methods

### 2.1. Evidence Acquisition

A search of the PubMed database was performed to identify original and review articles in English that address the anatomy of the prostate and relevant structures adjacent to the prostate, without any limit to publication date. The keywords used were levator ani muscle, rhabdosphincter, urethra, perineal membrane, smooth muscles, elastic fibers, continence, and pelvic floor anatomy. Relevant articles and textbook chapters were reviewed, analyzed, and summarized with the consensus of the authors of this paper.

### 2.2. Anatomical Study

The study was performed in accordance with the provisions of the Declaration of Helsinki 1995 (as revised in Edinburgh 2000) [15]. The histological sections shown in this review were newly prepared from 9 donated cadavers (6 men and 3 women) ranging in age from 76 to 95 years, with a mean age of 85 years. In addition to observations of the sections from these 9 cadavers, the results of the present study were ensured by observations of previously made histological sections from the other 54 cadavers (30 men and 24 women) which were dissected our previous studies [14, 16, 17]. Detailed information is shown in Table 1.

The cause of death had been ischemic heart failure or intracranial bleeding and we made sure that none of the individuals had undergone surgery by reference to medical documentation as well as macroscopic observation after opening the abdominopelvic cavity. None of the cadavers in the present study underwent radiotherapy. They also had no IBD, connective tissue, or muscular diseases when they were alive. These cadavers had been donated to Sapporo Medical University or Tokyo Dental College for research and education on human anatomy, and their use for research had been approved by the university ethics committees. The donated cadavers had been fixed by arterial perfusion with 10% v/v formalin solution and stored in 50% v/v ethanol solution for more than 3 months.

After routine procedures for paraffin-embedded histology, most sections were stained with hematoxylin and eosin (HE), azan, or silver staining, and some were used for immunohistochemistry as well as elastica-Masson staining (a variation of Masson-Goldner staining) or aldehyde fuchsin staining for elastic fibers (for the latter two staining methods, see Kawase et al. [13]). With reference to Hieda et al. [18] and Hinata et al. [19], the primary antibodies used for nerve immunohistochemistry were (1) mouse monoclonal anti-human S100 protein (1 : 200 dilution; Dako Z0311; Dako, Glostrup, Denmark), (2) rabbit polyclonal anti-human neuronal nitric oxide synthase (nNOS) (1 : 200; Cell Signaling Technology, Beverly, MA), (3) mouse monoclonal anti-human vasoactive intestinal peptide (H-6 or VIP) (1 : 100 dilution; Santa Cruz sc25347; Santa Cruz, CA), and (4) rabbit polyclonal anti-human tyrosine hydroxylase (TH) (1 : 100; Millipore-Chemicon ab152, Temecula, CA). With reference to Arakawa et al. [20] and Abe et al. [6], the antibodies used for immunostaining of fibrous structures were (1) mouse monoclonal anti-human desmin (dilution, 1 : 50; Dako) and (2) mouse monoclonal anti-human alpha smooth muscle actin (1 : 100; Dako M0851, Glostrup, Denmark). The secondary antibody was labeled with horseradish peroxidase (HRP), and antigen-antibody reactions were detected by the HRP-catalyzed reaction with diaminobenzidine. Counterstaining with hematoxylin was performed on the same samples. A negative control without a primary antibody was set up for each of the specimens. Observations and photography were usually performed with a Nikon Eclipse 80. Photos at ultralow magnification (objective lens less than ×2) were taken using a high-grade flat scanner with translucent illumination (Epson scanner GTX970).

#### 2.2.1. Connection between the Levator Ani and Rhabdosphincter in Males

**Figure 1:**
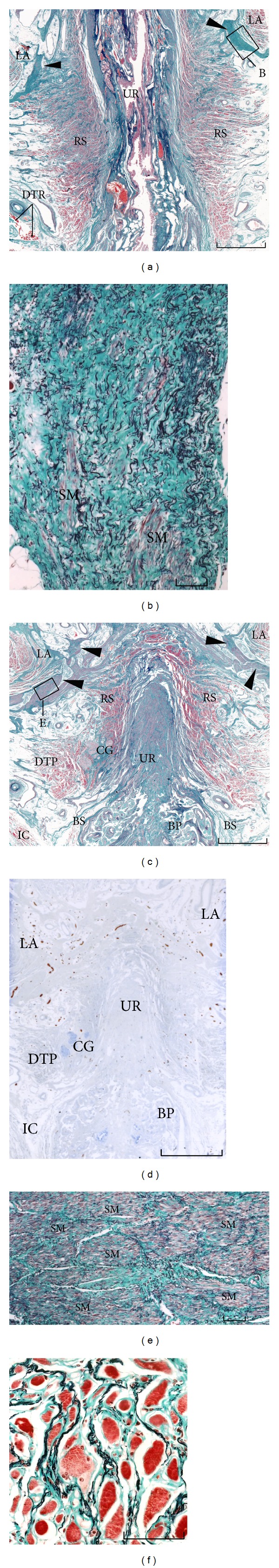
Frontal sections in a 78-year-old man. Panels (a)–(c) and (e) show elastica-Masson staining (striated muscles: red; collagen fibers: green; elastic fibers: black), while panel (d) shows immunohistochemistry for S100 protein using a section close to that in panel (c). Panel (a) includes a longitudinal section of the urethral wall (UR), while panel (c) is located 3 mm behind panel (a). Panel (b) (or panel (e)) is a higher-magnification view of the square in panel (a) (or panel (c)). A thick fascial structure (arrowheads in panels (a) and (c)), containing abundant elastic fibers (black wavy lines in panels (b) and (e)) and smooth muscles (SM; red-gray fibers in panels (b) and (e)), is evident between the rhabdosphincter area (RS) and the inferomedial edge of the levator ani (LA). In panel (d) (section adjacent to panel (c)), S100-positive nerves are seen passing through the urogenital hiatus. Panel (f), a higher-magnification view of the center of the rhabdosphincter area on the left-hand side of panel (a), displays an elastic fiber cage surrounding the striated muscle fibers. Scale bars: 10 mm in panels (a), (c), and (d); 0.1 mm in panels (b), (e), and (f). BP: bulbus penis; BS: bulbospongiosus muscle; CG: Cowper's gland; DTP: deep transverse perineal muscle; IC: ischiocavernosus muscle.

**Figure 2:**
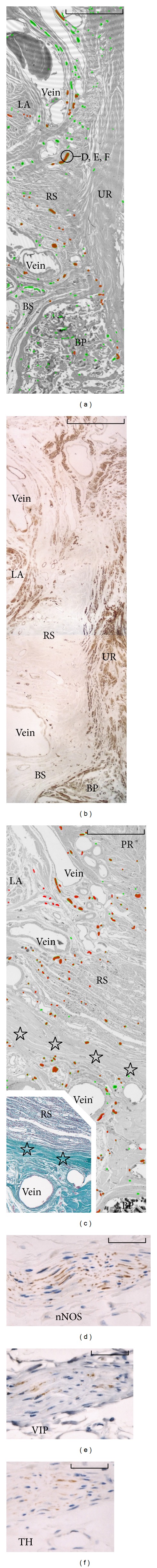
Frontal sections in an 84-year-old man. Panels (a) and (c) (nerve mapping), including a longitudinal section of the urethra (UR), display nerve distributions according to immunohistochemistry using sections adjacent to panels (a) and (c) (red color: nNOS-positive nerves; green color: the other nerves). Panel (c) is located 8 mm anterior to panel (a). Abundant nerves pass through the urogenital hiatus between the rhabdosphincter area (RS) and levator ani (LA). Stars in panel (c) indicate a solid, perineal membrane along the inferior margin of the rhabdosphincter area. Insert in panel (c) emphasizes the perineal membrane. Panel (b) (immunohistochemistry for smooth muscle actin) exhibits smooth muscles in and along the inferomedial edge of the levator ani (LA) in a section near that shown in panel (a). A nerve indicated by a circle in panel (a) is shown in panel (d) (nNOS immunohistochemistry), panel (e) (VIP), and panel (f) (tyrosine hydroxylase or TH). The nNOS-positive nerve, a candidate for the cavernous nerve, usually contains TH-positive fibers. Scale bars: 10 mm in panels (a), (b), and (c); 0.1 mm in panels (d), (e), and (f). BP: bulbus penis; BS: bulbospongiosus muscle; PR: prostate.

Frontal sections clearly demonstrate that, in the male, the bilateral slings of the LA sandwich the rhabdosphincter area (Figure 1). This topographical relationship strongly suggests that the LA provides mechanical support. In fact, it has been considered that the function of the LA to rapidly cut off urinary flow is effected via active elevation of the urethra, in contrast to the slow-twitch nature of the rhabdosphincter [21, 22]. Thus, it seems reasonable to assume that there are specific structures suitable for transduction of force from the LA to the urethral wall, via the rhabdosphincter area. A thick fascial structure has been reported to connect the rhabdosphincter area to the LA [10, 23]. This fascia or interface structure contains abundant elastic fibers and smooth muscles, which are irregularly arrayed (Figures 1(b), 1(e), and 2).

Shafik [24] provided a concept “the hiatal ligament” to the entire connective tissue occupying the urogenital hiatus, but this term is recently used for the perianal tissue (reviewed by Kinugasa et al. [25]). We do not deny a possibility that large urogenital hiatus shown in the present study is a result of degeneration of the LA with age [26]. In this case, to fill the developing space, the interface tissue between the LA and rhabdosphincter area may be enlarged with age.

**Figure 3:**
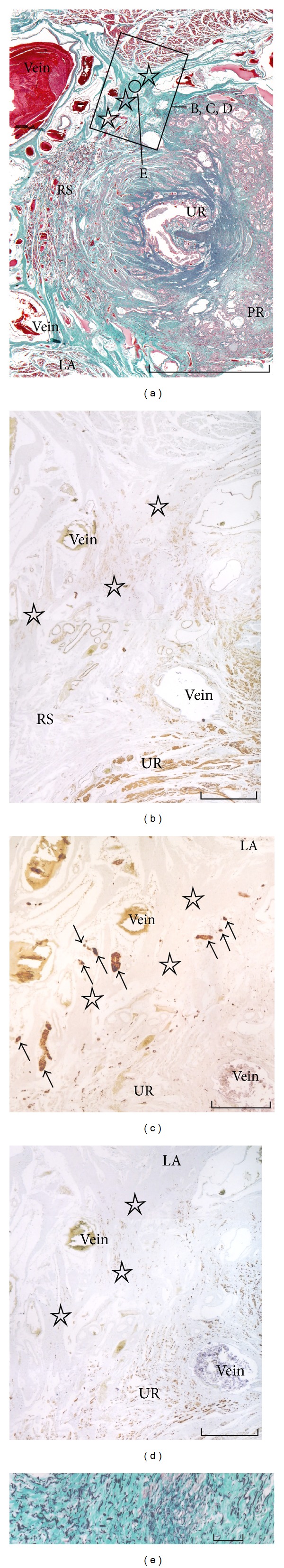
Horizontal sections in an 87-year-old man. Panel (a) (elastica-Masson staining) includes the urethra (UR), prostate (PR), rhabdosphincter (RS), and levator ani (LA): the anterior side of the body corresponds to the left-hand side of the figure. Panel (b) (immunohistochemistry for smooth muscle actin), panel (c) (immunohistochemistry for S100 protein), and panel (d) (immunohistochemistry for desmin) are higher-magnification views of the square in panel (a) and display thick fascia (stars) connecting the levator and rhabdosphincter areas. The connecting fascia contains abundant smooth muscle (panel (b)) and several nerves (arrows in panel (c)). Mature smooth muscle is positive for desmin (panel (d)). Panel (e), a higher-magnification view of the small area marked “E” in panel (a), exhibits irregularly arrayed elastic fibers in the connecting fascia. Scale bars: 10 mm in panel (a); 1 mm in panels (b), (c), and (d); 0.1 mm in panel (e).

The fascia or interface connecting the LA to the rhabdosphincter also contains veins and nerves, the latter originating from the periprostatic neurovascular bundle and passing through the urogenital hiatus toward the penile hilum (Figures 1(d), 2(a), and 2(c)). These autonomic nerves may be systematically classified into (1) tyrosine-positive sympathetic nerves, (2) neuronal nitric oxide synthase (nNOS)- or vasoactive intestinal polypeptide (VIP)-positive parasympathetic nerves, and (3) mixed nerves containing all of these fibers (Figures 2(d), 2(e), and 2(f)). However, Hieda et al. [27] reported that the majority corresponds to the double positive nerves (nNOS+, VIP−, TH+), especially in males, in contrast to a small number of the triple positive nerves (nNOS+, VIP+, TH+) and the single positive sympathetic nerves (nNOS−, VIP−, TH+). Thus, nNOS-positive nerve fibers are seen not only in the cavernous nerves but also most of pelvic plexus branches. Takenaka et al. [28] considered the interface area between the LA and rhabdosphincter to be the most likely route for the cavernous and sphincter nerves. Horizontal sections also demonstrate a connecting fascia between the LA and the rhabdosphincter (Figure 3) although clarification of this issue would be highly dependent on the superoinferior level of horizontal sectioning.

#### 2.2.2. Collagen Fibers and Elastic Fibers

Collagen fibers (type I collagen fibers) possess very little elasticity, whereas elastic fibers absorb tensile stress and recover their length. Tendons of skeletal muscles, which are composed of mostly type I collagen fibers, require a small proportion of elastic fibers in order to recover their length after muscle contraction [29, 30]. However, as typically seen in extremities, the covering fascia of a striated muscle contains few or no elastic fibers, because its elastic nature is due largely to the mesh structure of its constituent collagen fibers [31] and partly to the elasticity of the muscle cells themselves [32]. Fasciae that are exceptionally elastic fiber-rich cover or bundle striated muscle fibers in the extraocular muscles of the eye [33], the intrinsic lingual muscles along the tongue surface [34], and also the LA and external anal sphincter [20, 35]. Notably, all of these muscles are inserted into soft tissues, and not into bones.

**Figure 4:**
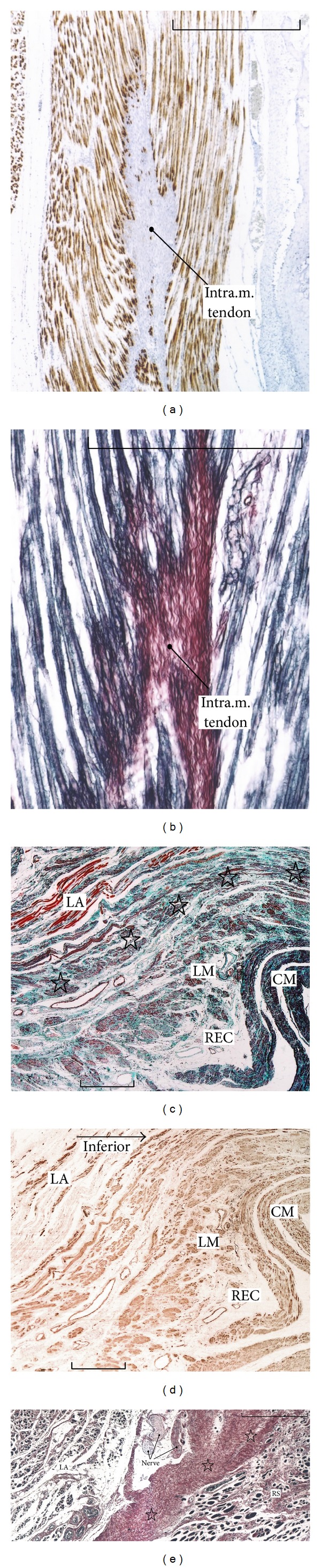
Comparative morphology of striated muscle insertions: the subscapularis muscle of the shoulder and the levator ani. Specimens obtained from a 95-year-old man. Panel (a) (immunohistochemistry for an intermediate filament, desmin) and panel (b) (silver staining) are near sections showing longitudinally sectioned subscapularis muscle fibers inserting into an intramuscular tendon (intra.m.tendon). Desmin spots along the tendon (panel (a)) correspond to anchor structures between the muscle and type I collagen fibers in skeletal muscles. In panel (b), type IV and other collagens (black) surrounding the muscle fibers are connected with type I collage (red) in the intramuscular tendon. Panel (c) (elastica-Masson staining) and panel (d) (immunohistochemistry for smooth muscle actin) are adjacent sections exhibiting the insertion of the levator ani (LA) to the lower rectum (REC). Longitudinally running striated muscle fibers insert into the conjoint longitudinal muscle coat of the rectum (stars). Panel (e) (silver staining) shows the inferomedial edge of the levator ani facing the rhabdosphincter (RS). Muscle fibers of both the levator and sphincter are cut transversely or obliquely, and thick fascia (stars) is interposed between these muscles. CM or LM, circular or longitudinal smooth muscles of the lower rectum. All scale bars: 1 mm.

**Figure 5:**
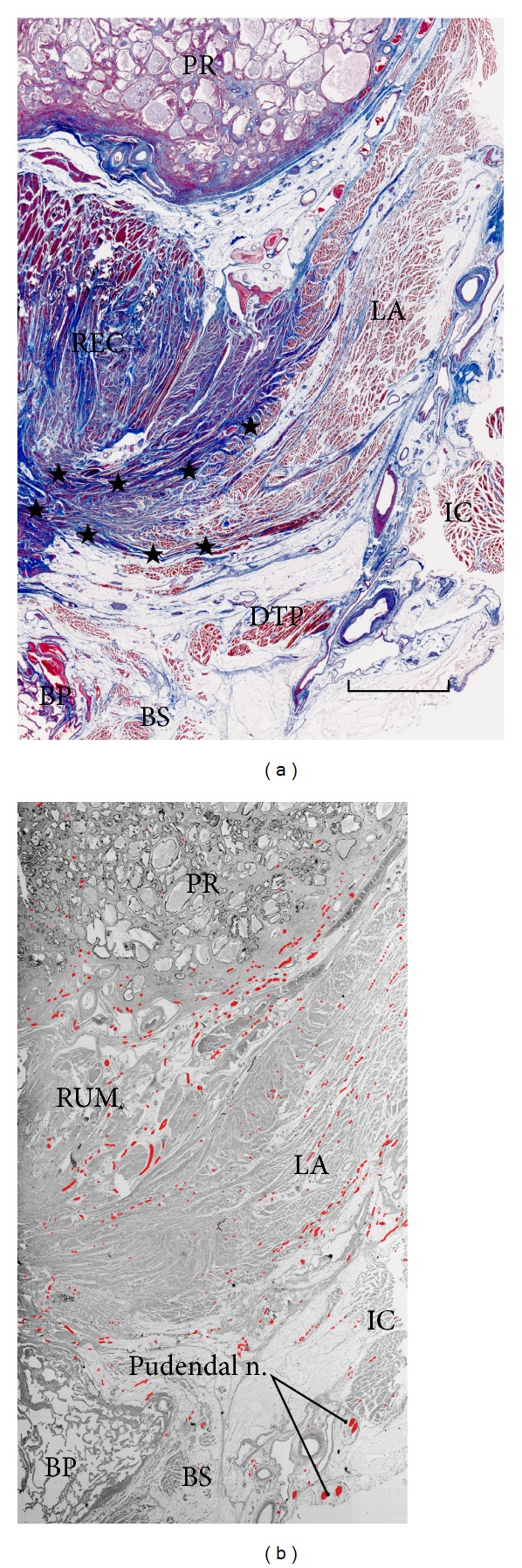
Levator ani insertion to the rectum seen in a frontal section of a 91-year-old man. Panel (a) (azan staining) displays the covering fasciae and intramuscular tendons (stars) of the levator ani (LA) converging into the longitudinal muscle layer of the anterior wall of the rectum (REC). Both striated and smooth muscle fibers are red, while connective tissues (collagen and elastic fibers) are dark blue. The definite fibrous connection between the levator ani and rectum is quite different from that between the levator and urethra. Panel (b) (nerve mapping according to S100 protein immunohistochemistry) exhibits a level 5 mm anterior to panel (a). The rectourethralis muscle (RUM) provides one of major pathways of the cavernous nerve from the periprostatic region to the penile hilum. These panels were prepared at the same magnification (scales bars: 10 mm in panel (a)). BP: bulbus penis; BS: bulbospongiosus muscle; DTP: deep transverse perineal muscle; IC: ischiocavernosus muscle; PR: prostate.

As typically seen in arterial walls, smooth muscles and elastic fibers usually coexist because elastic fibers are necessary for maintaining the 3-dimensional configuration of smooth muscle fibers [36]. As well as the vascular wall, in the pelvic floor, smooth muscle and elastic fibers coexist (Figures 1(e), 2(b), 3(b), and 3(e)). For the anorectum, the LA insertion provides a smooth muscle-rich connective tissue band that is continuous with the conjoint longitudinal muscle coat [20] (Figures 4(c) and 4(d)) or longitudinal anal muscle [37]. The interface smooth muscles are also continuous with the rectourethralis muscle [38] (Figure 5(b)). Nerves supplying the internal anal sphincter originate from the periprostatic neurovascular bundle, run downward, and penetrate the rectal wall along the conjoint longitudinal muscle [18]. This kind of elastic interface between a striated muscle and a soft tissue target would seem to minimize any damage or tears resulting from sudden and strong contraction of the LA. In this context, the connecting fascia between the LA and the rhabdosphincter would seem to play a role in (1) stabilizing structures in the event of elevation force and (2) regulating and distributing tensile stress from the LA.

#### 2.2.3. Muscle Fiber Direction and Force Transduction

In a skeletal muscle, muscle fiber contraction moves type IV collagen-rich endomysium, which is connected with a type I collagen-rich epimysium and intramuscular tendon (Figures 4(a) and 4(b)). Multiple leafs of the epimysium join together to provide an intramuscular tendon, which converges into an extramuscular tendon [3]. Although the composite fibers are different (Figures 4(c) and 4(d)), a tight connection between the LA and rectum is rather similar to the skeletal muscle morphology (Figure 5(a)). However, between the LA and rhabdosphincter, such gradual changes are not carried in collagenous structures but in a thick fascia or interface tissue containing randomly arrayed elastic fibers and smooth muscles (Figures 1(b), 1(e), 3(b), 3(d), 3(e), and 4(e)).

The most striking difference between the LA and other striated muscles lies in the relationship between the direction of muscle action and that of the muscle fibers: in skeletal muscle, the muscle fibers, tendon, and fibrous tissue connecting the two are consistently arranged “in series” along an almost straight line (Figures 4(a) and 4(b)), whereas the LA muscle fibers are not directed to the urethra or rhabdosphincter area (Figure 4(e)). Thus, the term “pubourethralis muscle” (the most anterior part of the LA [39]) may not indicate the function but merely the muscle location near the urethra.

**Figure 6:**
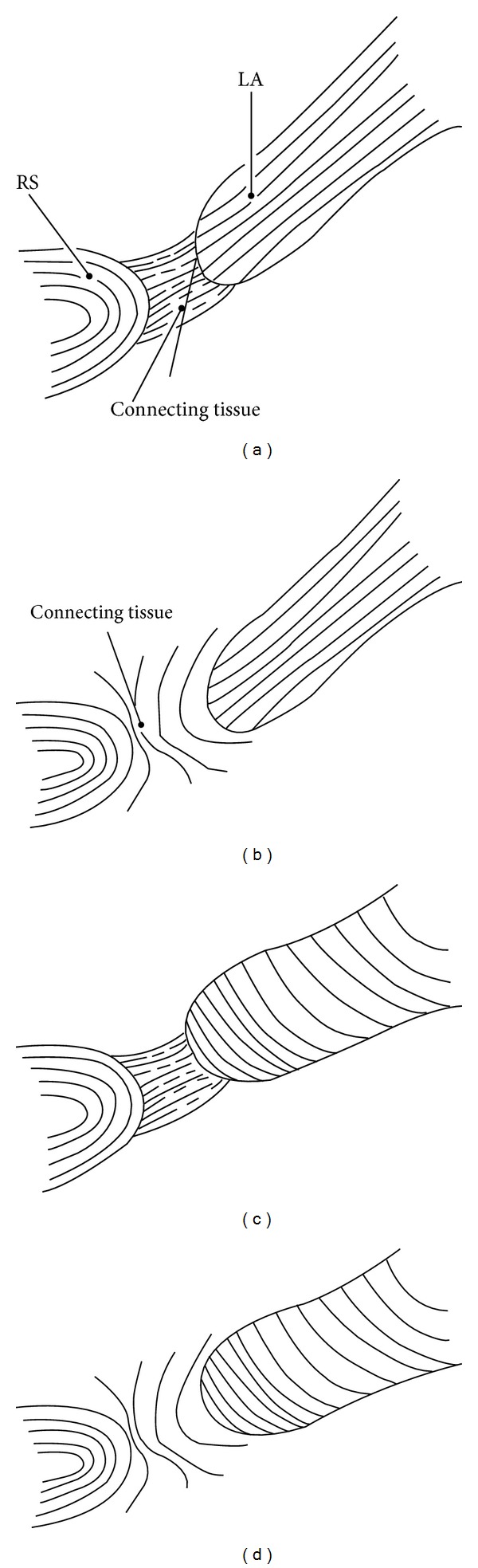
Diagram of interface tissue configurations between the rhabdosphincter and levator ani. In panels (a) and (b), muscle fibers of the levator ani directing inferomedially are suitable for upward traction of the rhabdosphincter area, whereas in panels (c) and (d), the fiber directions of the oblique muscle are not suitable for such traction. In panels (a) and (c), the fibrous tissues or fasciae connecting between the levator ani and rhabdosphincter are arranged in series along the axis from the levator to the urethra, whereas in panels (b) and (d), the fasciae along or surrounding the levator and/or the rhabdosphincter area are not suitable for force transduction but for sliding between these two muscles. The morphology shown in panel (a), being similar to the configuration between a skeletal muscle and bone when the connecting fibrous tissues are formed by collagenous fibers, is most suitable for upward traction. The levator and anorectum exhibit this type of morphology, but the connecting tissues are composed of elastic fibers and smooth muscle. The morphology shown in panel (b) is similar to that between the levator ani and external anal sphincter. Panel (c) displays the actual morphology including the levator ani and rhabdosphincter because the fibers of the levator ani muscle are directed to the anorectum, external anal sphincter, and anococcygeal ligament. However, the connecting fibrous tissues contain abundant elastic fibers and smooth muscle. Panel (d) corresponds to the configuration between the levator and vagina that allows mutual sliding.

There are likely four morphological combinations for striated muscle fibers and their target (Figure 6). Most skeletal muscles and their insertion sites show the type A morphology (Figure 6(a)), whereas the LA and rhabdosphincter area seems to exhibit the type C morphology (Figure 6(c)). Moreover, the rhabdosphincter muscle fibers are not bundled by collagen fibers but by elastic fibers (Figure 1(f)), and hyaluronic acid seems to act as a lubricant between the elastic fibers [14]. Such an elastic fiber cage for striated muscle fibers is also seen in intrinsic lingual muscles [34] and the external anal sphincter [20]. Consequently, the LA seems unsuitable for elevation of the urethra and rhabdosphincter: its action on the urethra might be overemphasized relative to that on the anorectum. The pelvic floor muscle contraction tended to be analyzed as a whole [40] and, thus, LA function on the urethra might not be compared with that on the anorectum.

#### 2.2.4. Smooth Muscles in Connective Tissue

We have already described the smooth muscles covering the inferomedial end of the LA, that is, the interface tissue between the LA and rhabdosphincter area. Do these smooth muscle fibers exert synchronized action with the LA under the control of nerve impulses? Smooth muscles in the pelvic floor connective tissue are not arrayed regularly and are directed at random. Therefore they are likely to differ in function from the regularly arrayed smooth muscle structure typically seen in the intestine. The latter type of structure shows organized contraction or peristalsis under the control of nerves and hormones, but smooth muscles in connective tissue may not, in view of their random arrangement. This is somewhat reminiscent of the nature of smooth muscle cells or fibers in the walls of arteries, which can act against blood pressure without nerve or hormonal control (i.e., Bayliss effect; increased pressure and subsequent stretch of smooth muscle causes muscle contraction and increased resistance [41–43]). Connective tissue composed of smooth muscle would seem to function as an ideal barrier, septum, or protector against mechanical stress because, even without innervation, smooth muscle fibers resist (not absorb) pressure in accordance with Bayliss effect. This function seems to be much stronger than the passive action of elastic fibers.

The “integrated pelvic floor theory” [44] attributed a key role to the longitudinal anal muscle (=conjoint longitudinal muscle coat; see Section 2.2.2) in the statics and dynamics of the pelvic viscera, being involved in the closure and opening of the urethra and anal canal. According to Petros [44], the smooth muscles, with their vertical course, create a downward force for bladder neck closure during effort and stretch the outflow tract open during micturition. However, we do not think that smooth muscles in the pelvic floor connective tissue play a strong, monodirectional, and long-term traction role without cooperation of striated muscle function because of their random arrangement and nonexistence of any nerve network such as the myenteric plexus for intestines. Moreover, the longitudinal muscles are located away from the female urethra or its sphincters but they end more posteriorly around the vagina and vaginal vestibule [45].

#### 2.2.5. Separation of the Rhabdosphincter from the LA by the Vagina and Paracolpium

**Figure 7:**
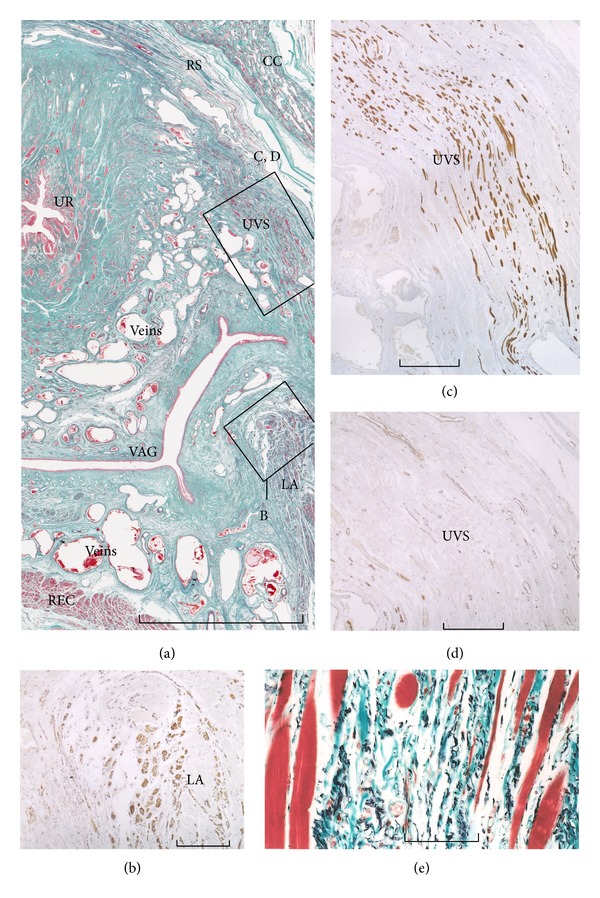
Horizontal sections in a 76-year-old woman. Panel (a) (elastica-Masson staining) includes the urethra (UR), crus clitoris (CC), rhabdosphincter (RS), urethrovaginal sphincter (UVS), and levator ani (LA): the anterior side of the body corresponds to the upper side of the figure. Panel (b) (immunohistochemistry for smooth muscle actin) is a higher-magnification view of the square marked B in panel (a), while panel (c) (immunohistochemistry for desmin) and panel (d) (immunohistochemistry for smooth muscle actin) are higher-magnification views of the square marked CD in panel (a). The levator ani is accompanied by a smooth muscle-rich fascia (panel (b)). The urethrovaginal sphincter is composed of striated muscle fibers (positive in panel (c); negative in panel (d)), accompanied by abundant irregular elastic fibers (panel (e)). The rectovaginal septum between the vagina (VAG) and rectum (REC) is replaced by veins. Scales bars: 10 mm in panel (a); 1 mm in panels (b), (c), and (d); 0.1 mm in panel (e).

Horizontal sections clearly demonstrated that the lateral part of the vagina is likely to be interposed between the rhabdosphincter area and the LA (Figure 7). Thus, in females, it seems difficult for the LA to attach (or even connect) to the rhabdosphincter area. Similar to the situation in males, the fasciae in and along the LA contain abundant smooth muscle (Figure 7(b)). The space surrounding the inferomedial edge of the LA, vagina, and rectum, that is, the inferior part of the paracolpium or the lower paracolpium, contains abundant nerves and ganglion cell clusters [27] and is continuous with the mesorectum, as Denonvilliers' fascia or the rectovaginal septum is fragmented or unclear. The LA carries no specific interface structure to insert into the vagina and is simply attached to the latter [20] although upper part of the vagina is connected with the endopelvic fascia lining the levator ani by two elastic fasciae, that is, the pubocervical fascia and the rectovaginal septum [16, 46].

Donker [47] appears to have been the first researcher to have noted abundant autonomic nerves running through the paracolpium from the pelvic plexus to the urologic organs, and his observations were collaborated by Ball Jr. et al. [48]. Thus, the paracolpium is likely to include part or most of the pelvic autonomic nerve plexus. Using late-stage fetuses, Kato et al. [49] demonstrated the female cavernous nerve running through the putative paracolpium. Hirata et al. [50] provided a beautiful diagram of the course of the female cavernous nerve covered by the pubocervical fascia, and, later, Hinata et al. [19] provided a series of photographs demonstrating the morphology.

In contrast to the relatively solid fascia lying between the LA and rhabdosphincter area in males, the female urethral sphincter was separated from the LA not only by the loose paracolpium but also by the vaginal lateral edge. However, the female urethra is tightly connected with and supported by the vagina [51]. This tight connection is formed at the fetal stage as a result of vaginal descent occurring in combination with reconstruction of the posterior wall of the urethra [52]. On this basis, Hinata et al. [16] have postulated the optimal surgical approach to this tight connection.

#### 2.2.6. Female Rhabdosphincter and Perineal Membrane

The gender difference in the morphology of the rhabdosphincter is well known, being omega-shaped in males and having a semicircular configuration in females [10, 53, 54]. In early fetuses, the female rhabdosphincter is unable to extend inferoposteriorly because of the developing vestibule close to the sphincter [55]. The anteriorly restricted, female rhabdosphincter may require a static ligament-dependent lateral support (i.e., the perineal membrane; see below in Section 2.2.6), in contrast to the dynamic muscle-dependent support in males.

As very poorly developed rhabdosphincter is reportedly predominant in Japanese elderly women, Kurihara et al. [56] considered the thick, longitudinal smooth muscle layer of the female urethra to play a major role in continence. Hirata et al. [35] reported a gender difference in the fiber architecture of the endopelvic fascia (fascia pelvis parietalis; a fascia lining the superior or inner aspect of the LA): the male endopelvic fascia is multilayered and contains abundant smooth muscle fibers, whereas the female endopelvic fascia is solid, thick, and contains abundant elastic fibers rather than smooth muscle. Such a difference in connective tissue may be the result of the different hormonal backgrounds, as estrogen is known to increase the formation of elastic fibers [57].

According to Oelrich [58], the female rhabdosphincter carries two extensions: a short superolateral extension (i.e., the compressor urethrae) and a long inferolateroposterior extension (i.e., the urethrovaginal sphincter). The urethrovaginal sphincter is easily to demonstrate in horizontal sections [56, 59], although Figure 7 shows a level slightly higher than the thickest part.

**Figure 8:**
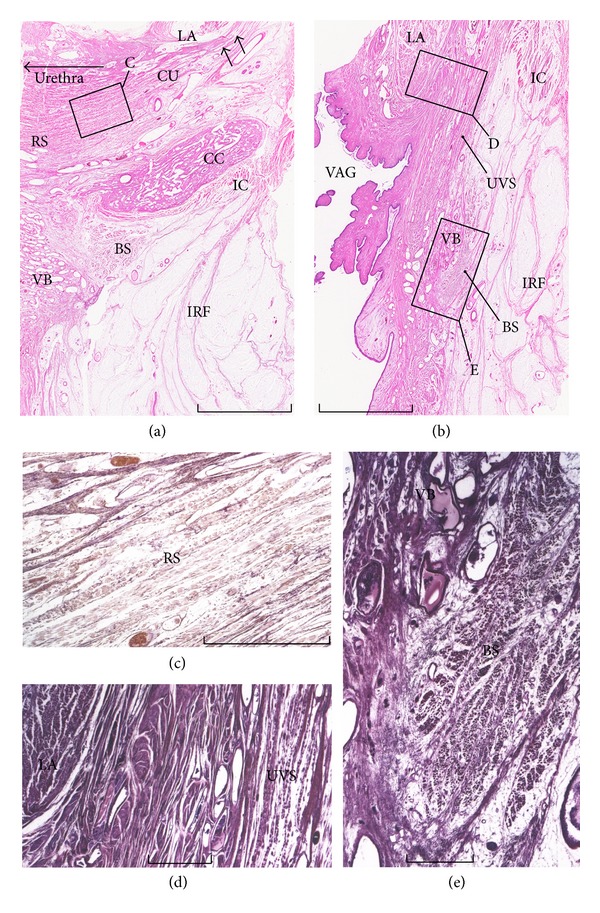
Frontal sections in an 83-year-old woman. Panels (a), (b), (d), and (e): HE staining; panel (c): aldehyde fuchsin staining (elastic fibers, violet). In panels (a) and (c), elastic fibers between the muscle fibers of the rhabdosphincter (RS) join to provide a thick fascial structure, the perineal membrane (arrows in panel (a)). The levator ani is attached to the upper aspect of the membrane. Panel (b), 25 mm behind panel (a), includes the posterior end of the vestibular bulb (VB). Part of the rhabdosphincter, the urethrovaginal sphincter (UVS), extends in and along the perineal membrane in panel (b). Scale bars: 10 mm in panels (a) and (b); 1 mm in panels (c), (d), and (e). BS: bulbospongiosus muscle; CC: crus clitoris; CU: compressor urethrae; DTP: deep transverse perineal muscle; IC: ischiocavernosus muscle; IRF: ischiorectal fossa; VAG: vagina; VB: vestibular bulb.

Despite continuity with the major part of the rhabdosphincter, the compressor urethra is difficult to demonstrate because it is restricted to a relatively thin frontal plane (Figure 8(a)) and because well-developed examples were very small in number [49]. In the horizontal plane, the compressor is difficult to discriminate from the urethrovaginal sphincter [59]. Using histology and macroscope slices, Stein and DeLancey [60] found that both the compressor urethrae and urethrovaginal sphincter are closely “associated” with the female perineal membrane. However, in their photos, it was difficult to identify the composite fibers of the perineal membrane. Moreover, unlike our interpretation, they considered the perineal membrane to connect with the endopelvic fascia.

Kato et al. [49] clearly demonstrated that elastic fibers between the rhabdosphincter muscle fibers join together to form the perineal membrane. Thus, an elastic fiber cage for the rhabdosphincter muscle fibers (Figure 1(f)) is a common feature in both genders, although the male perineal membrane is solid, collagen fiber-rich, and extends along the inferior margin of the rhabdosphincter area (Figure 2(c) insert).

Mirilas and Skandalakis [61] simply considered the perineal membrane as a structure extending between the bilateral LA slings, but it is similar to a concept of the hiatal ligament by Shafik [24]. In females, striated muscle fibers of the compressor urethrae are embedded in the elastic fiber mesh of the perineal membrane immediately superior to the crus clitoris (Figure 8(a)). Kato et al. [49] demonstrated that cavernous nerve candidates cross the perineal membrane near the compressor urethrae from the intrapelvic to the extrapelvic space.

The perineal membrane extends medially and inferiorly along the vaginal wall (in which the urethrovaginal sphincter is embedded) and changes into a solid membrane between the vestibular bulb and vaginal wall (Figure 8(b)). The LA is located in the superior, medial, and posterior sides of the perineal membrane. Thus, in females, the inferomedial edge of the LA does not attach to or even face the rhabdosphincter area. Instead, the LA seems to act on the rhabdosphincter and urethra via the perineal membrane attached to the LA. However, such an indirect connection between the LA and female rhabdosphincter seems not to contradict the evidence by Morgan et al. [62] that the LA acts more strongly to the longer female urethra.

#### 2.2.7. Deep Transverse Perineal Muscle

**Figure 9:**
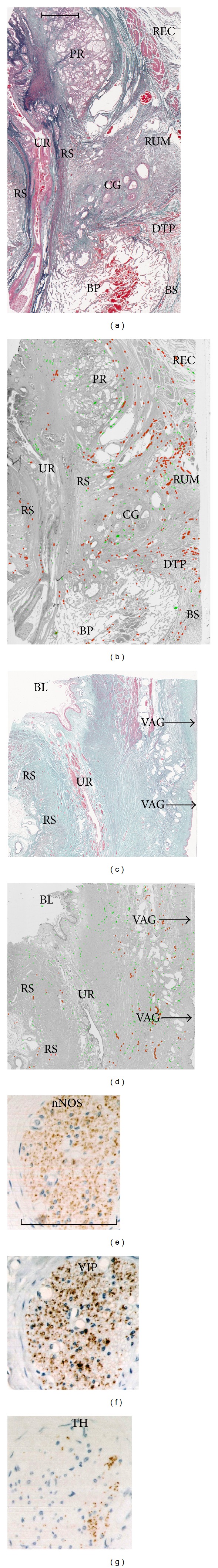
Sagittal sections in a 91-year-old man and an 88-year-old woman. Panel (a) (azan staining) and panel (c) (elastica-Masson staining) display the urethra (UR) and rhabdosphincter area (RS) in a 91-year-old man and an 88-year-old woman, respectively. In panel (a), the rectourethralis muscle (RUM; smooth muscle) is seen between Cowper's gland (CG) and the rectum (REC). Panel (c) includes the urethral opening to the bladder (BL). Panels (b) and (d) (nerve mapping) display nerve distributions according to S100 protein immunohistochemistry using sections adjacent to those in panels (a) and (c). In both genders, abundant nerves are evident on the posterior side of the urethra (UR). Panel (e) (nNOS immunohistochemistry), panel (f) (vasoactive intestinal polypeptide: VIP), and panel (g) (tyrosine hydroxylase: TH) are higher-magnification views of a nerve near the vaginal wall (VAG). The abundant VIP-positive fibers characterize female pelvic nerves. Panels (a)–(d) ((e)–(g)) were prepared at the same magnification. Scale bars: 10 mm in panel (a) and 0.1 mm in panel (e). BP: bulbus penis; BS: bulbospongiosus muscle; DTP: deep transverse perineal muscle; PR: prostate.

Nakajima et al. [63] have reported that the deep transverse perineal muscle is attached to Cowper's gland in males and is continuous with the rhabdosphincter (Figures 1(c), 5(a), and 9(a)). With regard to the situation in females, although Fritsch et al. [64] reported that the muscle was absent, we believe that it is present, although vestigial, adjacent to Bartholin's gland. Thus, in both genders, the muscle is located inferiorly or superficially relative to the perineal membrane and not embedded in the membrane (see Section 2.2.6).

Likewise, a fascia covering the deep transverse perineal muscle may be regarded as the perineal membrane. Actually, because the male rhabdosphincter is continuous with the transverse muscle, the male perineal membrane along the bottom of the rhabdosphincter area (Figure 2(c) insert) is likely to attach to the muscle. The deep transverse perineal muscle has long been considered as a core of the urogenital diaphragm: the concept of the urogenital diaphragm will be discussed in the final part of this review.

#### 2.2.8. Rectourethralis Muscle

The rectourethralis muscle (smooth muscle mass in males) has been one of major interests in uroanatomy [65, 66], but its topographical relation with the LA was not described well. Frontal sections clearly demonstrate the rectourethralis muscle occupying the urogenital hiatus [38] (Figure 5(b)). Unlike the descriptions by Walz et al. [67], the rhabdosphincter is unlikely to attach to Denonvilliers' fascia because the rectourethralis muscle, to various degrees among individuals, is consistently interposed between the fascia and the sphincter [38, 68, 69].

In males, Rocco et al. [70] considered that the “tendinous” median dorsal raphe of the rhabdosphincter acts as a fulcrum for contraction. However, it is not tendinous or collagenous but composed of smooth muscle and elastic (not collagen) fibers. The tendinous nature of the dorsal raphe may have been overestimated through comparison with general morphology in experimental animals such as rats that carry the typical raphe [71]. Actually, a recent big review of the rhabdosphincter physiology was based on the rat anatomy [72]. The median part is continuous with the rectourethralis muscle or, rather, it is a part of the rectourethralis muscle [38]. Because of Bayliss effect, the smooth muscle may resist traction from the rhabdosphincter and urethra. Some urologists believed that the LA contributes to a double-sling mechanism via the median dorsal raphe for closure of the urethra in males [39, 73], but the LA does not extend to the midsagittal area (Figure 9(a)). This theory seems to be an analogy of the triple loop system of rhabdosphincter around the anal canal [74].

On the inferior side of the pelvic floor in males, the rectourethralis muscle is attached to the perineal body [69], whereas in females the superolaterally located perineal membrane is not attached to the perineal body. In fact, the perineal body in females is the thickest in the lateral part (not in the midsagittal plane) and contains abundant smooth muscle. However, it connects the external anal sphincter to the vagina [75]. Thus, Denonvillier's fascia does not seems to contribute to urethral support because it ends at the rectourethralis muscle in males (Figures 5(b) and 9(a)), whereas it is fragmented or absent in females. In the pelvic floor of females, the rectovaginal septum or Denonvilliers' fascia is a rare example of a structure composed of “regularly” arrayed elastic fibers [76].

In both genders, sagittal sections show that nerves are distributed much more densely on the posterior side of the rhabdosphincter than on the anterior side (Figures 9(b) and 9(d)): this nerve-rich area corresponds to the anterior, minor part of the paracolpium in females [19], and the rectourethralis muscle in males (Figures 9(a) and 9(c)). A nerve running longitudinally along the vagina may be included [77]. As Hoyle et al. [78] reported, nerves positive for VIP are richly distributed near and along the vaginal wall (Figure 9(f)). Tekenaka et al. [79] considered the rectourethralis muscle mass to be one of the multiple courses of the cavernous nerve.

#### 2.2.9. Nerves Passing through the Interface Tissues for the LA

We have described nerves passing through the perineal membrane, the rectourethralis muscle, and the interface tissue between the LA and rhabdosphincter area (Figures 1(d), 2(a), 2(c), 3(c), 4(e), 5(b), 9(b), and 9(d)). Most of these nerves take a long course from the pelvic plexus to the cavernous tissue, rhabdosphincter and urethral wall smooth muscles. Major targets of the nerves are not the interface tissues themselves: smooth muscles as a connective tissue do not need nerve supply according to Bayliss effect. We can remind an anatomical rule of the long nerve courses along striated muscles in the body: they run through the intermuscular septum (a definite fascial space) that provides a neutral zone free from tensile stress between a pair of striated muscle groups with different function (e.g., the flexors and extensors). Likewise, in the pelvic floor, such a pair of muscles seems to be found in the LA and rhabdosphincter, the LA and another perineal muscle and, the LA and longitudinal rectal muscle although the last one is not striated. These interfaces should conduct muscle contraction force, but not to damage the long nerves involved, elastic nature of the interface should make tensile stress weak and constant. This is an essential contradiction required for the pelvic floor interface tissues.

Urologists expect the active LA function to elevate the rhabdosphincter, but they do not want injury of nerves passing through the interface between the LA and sphincter area. For improved postoperative continence, the nerve-rich tissue seems to require a technique with athermal division and selective suture ligation of the dorsal vasculature complex without either bunching the vasculature or incision of the endopelvic fascia [80]. Surgical incision with bleeding makes a collagenous scar to connect the separated tissues: Oguma et al. [81] demonstrated the typical morphology after surgery although it was an experimental tendon incision, not in the pelvic floor. Gynecologists may not want impairment of elastic nature of the pelvic floor interface tissues. However, a polypropylene mesh against pelvic organ prolapse seems to be inserted into the nerve-rich interface tissues [82].

#### 2.2.10. Classical Schemes of the Pelvic Floor and the Levator Ani

**Figure 10:**
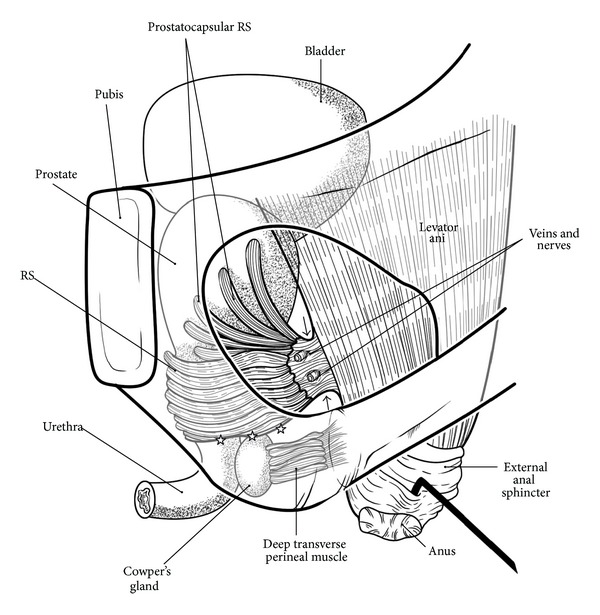
A diagram of the levator ani and rhabdosphincter in men. Lateral view through the bony pelvis. In the male, the rhabdosphincter is connected with the inferomedial margin of the levator ani. The fibers of the levator ani muscle are directed to the external sphincter and anus. By a thick fascia (sandwiched by arrows) containing veins, nerves, elastic fibers, and smooth muscle, the rhabdosphincter area in the male is connected with the inferomedial margin of the levator ani. Stars indicate a layer for the male perineal membrane (not drawn; see Figure 2(c) insert). On the lower side of the rhabdosphincter area, the deep transverse perineal muscle connects Cowper's gland and the internal surface of the ischiadic bone.

**Figure 11:**
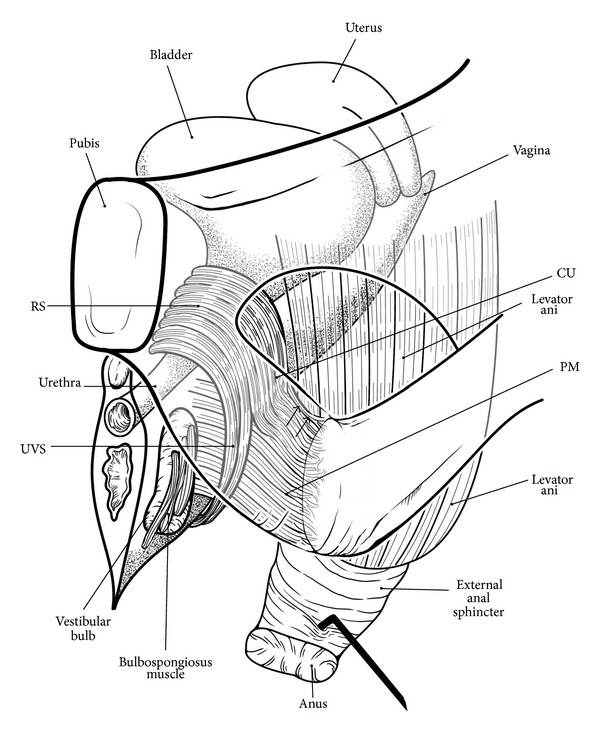
A diagram of the levator ani, rhabdosphincter, and perineal membrane in women. Lateral view through the bony pelvis. In the female, the rhabdosphincter is connected with the ischiadic bony ramus by the perineal membrane. The fibers of the levator ani muscle are directed to the external sphincter and anus. In the female, the rhabdosphincter is continuous with the anterior long muscle sheet, the urethrovaginal sphincter (UVS), and the posterolateral small part, the compressor urethrae (CU). These striated muscles are embedded in an elastic-fiber mesh that provides the insertion for the perineal membrane (PM) to the ischiadic bony ramus. The perineal membrane extends anteromedially to a space between the vestibular bulb and the vaginal wall. The levator ani is attached to the superior aspects of the perineal membrane (arrows).

To consider the gender difference in pelvic floor anatomy, it is pertinent to refer to the well-known schemes proposed by Oelrich [58, 83]. However, his schemes do not include the LA, and the viewpoints for the diagrams differ between the genders. Therefore we propose a revision of Oelrich's schemes, although the elegance of his original drawings may be lost in view of our limited technical skill (Figures 10 and 11). Oelrich [83] described large, upward extensions of the rhabdosphincter along the prostatic capsule. Later, these extensions were termed the “prostatocapsular rhabdosphincter” [84, 85]. We also emphasize the muscle extension (Figure 10).

In our diagrams, the interface between the rhabdosphincter and the LA is seen through the obturator foramen of the bony pelvis (Figures 10 and 11). For this view, however, the interface is shifted more posteriorly than the actual “lateral” position. Thus, the interface tissue might be mistakenly considered to occupy a position similar to that of the rectourethralis muscle. Actually, Uchimoto et al. [38] regarded the smooth muscle-made interface to the LA as an extension of the rectourethralis muscle.

The perineal membrane in the male is omitted in order to avoid confusion with neighboring lines. Likewise, in Figure 11 for the female pelvis, because the interface between the perineal membrane and the LA was difficult to draw, the LA is shifted from the actual superoposterior side of the perineal membrane to the posterior side. We have also omitted the ischiocavernosus muscle in both genders (see Figures 1(c), 5, and 8(a)), as well as the bulbospongiosus muscle in males (see Figures 1(c), 2(a), and 5).

#### 2.2.11. Urogenital Diaphragm

Finally, it is necessary to discuss the concept of the urogenital diaphragm, which is believed to be composed (simply) of the deep transverse perineal muscle. On the basis of magnetic resonance imaging studies, the strictest argument against the existence of the urogenital diaphragm was provided by Myers [86], who established a safe treatment for the retropubic veins in radical prostatectomy. He stated that “there is not a hint of what might be called Henle's artifact, his diaphragma urogenitale.” In spite of his excellent schemes, which included a membranous structure at the external genitalia, Oelrich [58, 83] also had a negative, rather than positive, opinion, because he did not identify striated muscles but smooth muscles in the membranous structure.

Nakajima et al. [63] considered that, because the deep transverse perineal muscle is not sheet-like but a 3-dimensional pillar continuous with the rhabdosphincter, previous researchers had found it difficult to identify, especially in histology preparations. The perineal membrane might also be considered a “fascia” of the transverse perineal muscle.

During dissection of the urogenital hiatus from the ischiorectal fossa on the anterior side of the rectum and on the lateral side of the urethra or vagina, we were able to touch by hand a diaphragm-like structure containing (1) the rhabdosphincter, its extensions, and the elastic interface tissue with the LA, (2) the perineal membrane, (3) the rectourethralis muscle, (4) the deep transverse perineal muscle, and/or (5) parts of the bulbospongiosus and ischiocavernosus muscles. Histological evidence for all of these structures has been provided in the form of photos in this review. Likewise, for measurement of thickness using clinical imaging, Betschart et al. [87] considered the perineal membrane as en mass that includes striates muscle, smooth muscle, and connective tissues. Because the deep transverse perineal muscle is sometimes thick and wide, even in elderly men [63] (Figure 5(a)), we do not need to rule out the concept of the urogenital diaphragm.

## 3. Concluding Remarks

For anorectal function, the LA muscle fibers are directed to and inserted into the upper rectum via a specific interface tissue, the conjoint longitudinal muscle coat. This feature is quite different from the other striated muscles. Although we cannot rule out a partial contribution of the LA to maintenance of continence, the LA seems unsuitable for pulling up the male urethral sphincter in view of the direction of the muscle fibers and because of the elastic fiber-rich interface with the sphincter area. Moreover, abundant nerves and veins pass through the interface. The interface tissue with the LA also contains abundant smooth muscle. Randomly arrayed smooth muscles in the pelvic floor, including those in the conjoint longitudinal muscle coat, are likely to act as a barrier, septum, or protector against mechanical stress because, even without innervation, they would resist pressure in accordance with Bayliss effect.

The male perineal membrane is thin but solid and extends along the inferior margin of the rhabdosphincter area. In contrast, in females, the rhabdosphincter, compressor urethrae, and urethrovaginal sphincter are embedded in the elastic fiber mesh that is continuous with the perineal membrane. The inferomedial edge of the female LA is attached to the upper surface of the perineal membrane and not directly attached to the rhabdosphincter. Thus, in female, the action of the LA on the urethral sphincter seems to be much less marked than in males. The urogenital diaphragm is not composed simply of the deep transverse perineal muscles but of multiple fibrous structures.

To understand the diverse structures concerning male and female urinary continence, we have provided an overview of the complex anatomy of the urethral rhabdosphincter, levator ani, and those surrounding structures. Knowing this anatomy and respecting, it should modify surgical procedures of radical prostatectomy or radical cystectomy and improve postoperative voiding and storage disorders. The present study has a limitation that the present materials did not include younger materials. Nevertheless, the because the patients undergo these surgeries are same ages with the cadavers donated for the presented study.

## Figures and Tables

**Table 1:** Specimens observed in this review.

	Male	Female	Ages (mean)	Donation
Newly prepared histological sections	6	3	76–95 (85)	TDC*
Sections from Hinata et al. (2013 JU elast) [14]	10	4	53–89 (74)	TDC
Sections from Hinata et al. (2012 JU cyst) [16]	0	20	64–88 (81)	TDC
Sections from Takenaka et al. (2005 Urol) [17]	20	0	65–92 (83)	SMU**

Total	36	27	53–95 (81)	

*TDC: donation to Tokyo Dental College.

**SMU: donation to Sapporo Medical University when the second author was the Professor and Head of the Department of Anatomy.
